# Role of Pseudo-Continuous Arterial Spin Labeling and 4D MR Angiography in the Diagnosis of Neck Paragangliomas

**DOI:** 10.3390/jcm14134725

**Published:** 2025-07-03

**Authors:** Andrea Romano, Allegra Romano, Giulia Moltoni, Serena Palizzi, Andrea Muscoli, Silvia D’Eufemia, Emanuela Parri, Antongiulio Faggiano, Alessia Bernardo Ciddio, Alessia Guarnera, Giacomo Suma, Alessandro Bozzao

**Affiliations:** 1 Department of Neurosciences, Mental Health and Sensory Organs, School of Medicine and Psychology, “Sapienza” University, 00185 Rome, Italy; allegra.romano@uniroma1.it (A.R.); giulia.moltoni@uniroma1.it (G.M.); serena.palizzi@uniroma1.it (S.P.); andrea.romuscoli@uniroma1.it (A.M.); silviadeufemia@yahoo.it (S.D.); emanuela.parri@uniroma1.it (E.P.); guarneraalessia@uniroma1.it (A.G.); alessandro.bozzao@uniroma1.it (A.B.); 2 Department of Clinical and Molecular Medicine, School of Medicine and Psychology, “Sapienza” University, 00185 Rome, Italy; antongiulio.faggiano@uniroma1.it; 3 Madonna della Fiducia Clinic, 00179 Rome, Italy; ciddietto@yahoo.it (A.B.C.); giacomosuma@fiducia.it (G.S.)

**Keywords:** pcASL, paraganglioma, schwannoma, TRICKS, 4D MR angiography, neck

## Abstract

**Background/Objectives:** The purpose of this study was to identify the most effective MRI techniques for detecting and characterising neck paragangliomas (NPGLs), which are highly vascularised tumors. **Methods:** Five readers were asked which MRI sequence among T2-WI, contrast-enhanced fat-suppressed T1-WI, pcASL, and TRICKS made them most confident in diagnosing paraganglioma. To evaluate concordance among the readers, the Fleiss K value was calculated. Sensitivity, specificity, and negative predictive values were calculated for each observer separately, and from all values, a mean was calculated. **Results**: The final cohort consisted of 28 patients (11 diagnosed with head-and-neck paragangliomas (HNPGLs)) of whom 7 were histologically confirmed and 4 identified based on a positive family history; 11 patients were undergoing familial screening (8 with HNPGLs and 3 without), and 6 patients had surgically confirmed vagal schwannomas. None of the schwannomas showed any increase in signal on pcASL sequences or arterial enhancement on TRICKS acquisition. The best concordance among readers was reached for pcASL and combined pcASL-TRICKS images (K = 1). **Conclusions:** The combined use of pcASL and TRICKS should be considered essential in a standardised protocol for characterising NPGLs.

## 1. Introduction

A variety of pathologies can affect the head and neck (HN) region, including neoplastic, infectious, inflammatory, and congenital lesions [1]. Among these, paragangliomas pose a particular diagnostic challenge, especially when a differential diagnosis with other solid neck masses is required.

Neck paragangliomas (NPGLs) are rare, slow-growing, hyper-vascular neuro-endocrine tumours that arise from paraganglionic tissue associated predominantly with the parasympathetic nervous system [1]. They most commonly occur, in descending order of frequency, at the carotid bifurcation, within the jugular foramen, in the tympanic cavity, and along the vagus nerve [2].

Imaging plays a key role both in the work-up of patients who present with a palpable neck mass and in the screening of asymptomatic individuals with a family history of paragangliomas. Ultrasound (US) is often the first-line modality because it is inexpensive and widely available; Doppler US and contrast-enhanced US (CEUS) improve vascular assessment, although they may be limited when evaluating deep-seated lesions [3].

Computed tomography (CT) performed before and after intravenous iodinated contrast is highly informative for head-and-neck lesions, but the use of ionising radiation and iodinated contrast medium may limit its applicability in some patients [4].

Magnetic resonance imaging (MRI) is the most sensitive modality for evaluating HNPGLs thanks to its excellent soft-tissue contrast and its ability to delineate intracranial extension. Larger lesions typically display a “salt-and-pepper” appearance caused by numerous vascular flow voids (“pepper”) interspersed with foci of sub-acute haemorrhage (“salt”) [1,5]. After gadolinium administration, the tumours usually show rapid, intense, homogeneous enhancement that reflects their rich vascularity [5].

Digital subtraction angiography (DSA) remains the reference standard for both diagnosis and pre-operative planning. It provides detailed haemodynamic information and directly visualises the early arterial enhancement that is characteristic of hyper-vascular tumours [6,7]. However, because DSA is invasive, there is growing interest in non-invasive MRI–based vascular techniques, including four-dimensional, time-resolved contrast-enhanced MR angiography (4D-CE-MRA) and arterial spin labelling (ASL). These sequences permit comprehensive evaluation of vascular dynamics and tumour perfusion and may reduce the need for invasive angiography in selected cases [8,9,10].

Time-resolved CE-MRA has shown excellent agreement with DSA for assessing tumour haemodynamics [11], whereas pseudocontinuous ASL (pcASL) can readily distinguish hyper-vascular from non-hyper-vascular lesions as recently demonstrated in skull-base tumours [12].

In the present study, we compared conventional and advanced MRI sequences—fat-suppressed, T2-weighted, fast spin-echo (FS-T2); contrast-enhanced, fat-suppressed, T1-weighted (CE-FS-T1); 4D-CE-MRA; and pcASL—evaluated individually and in combination for their ability to detect and characterise head-and-neck paragangliomas. Our aim was to identify the sequence or sequence combination that affords the highest diagnostic performance.

## 2. Materials and Methods

From January 2016 to January 2024, we prospectively enrolled patients who met all of the following criteria:

1. Known or suspected head-and-neck paraganglioma (HNPGL):
  ○ Individuals with a family history, with or without prior surgery or radiotherapy;
  ○ Subjects undergoing screening because of familial HNPGLs;
  ○ Patients with neck masses but no known familial predisposition;

○ MRI performed on a 1.5 T scanner (Signa Voyager, GE Healthcare, Chicago, IL, USA) that included
○ Conventional, fat-suppressed, T2-weighted fast spin-echo (FS-T2) and post-contrast, fat-suppressed, T1-weighted (CE-FS-T1) sequences;
○ Advanced sequences: pseudo-continuous arterial-spin-labelling (pcASL) and four-dimensional, contrast-enhanced MR angiography (4D-CE-MRA and TRICKS).

Exclusion criteria:

- Incomplete MRI protocol.
- Severe motion artefacts precluding image interpretation.
- Patients with base of the skull lesions (including PGLs).

### 2.1. Advanced Sequence Parameters

- 3D pcASL (acquired before contrast):

○ Labelling duration 1800 ms; post-labelling delay 2025 ms;
○ 3D stack-of-spirals, fast spin-echo read-out (eight spirals × 512 points);
○ FOV 24–26 cm; slice thickness 4 mm; in-plane resolution 3.6–4.5 mm^2^;
○ TE/TR 10.9/4840 ms; bandwidth 62.5 kHz; acquisition time 4–5 min;
○ A rapid phase-contrast angiogram of the neck vessels was obtained to position the labelling plane;

- 4D-CE-MRA (TRICKS):

○ One mask phase followed by 20 dynamic phases during injection of a gadolinium agent (4 mL s^−1^) and a 15-mL saline flush;
○ TR/TE 3.3/1.3 ms; flip angle 20°; FOV 24 cm; matrix 220 × 212;
○ Slice thickness 1.6 mm (36-partition slab, sagittal orientation);
○ Temporal resolution 1.8 s per phase, total 37.8 s;
○ Automatic subtraction of the mask to suppress the background signal.

### 2.2. Image Interpretation

Five neuroradiologists with 3–20 years of experience (A.B., G.T., F.T.C., G.M., and A.R.) independently reviewed all examinations. Clinical data were withheld, and studies were randomised.

Readers answered two questions for each study:

1. Presence (present | indeterminate | absent) and site of a lesion.
2. Sequence(s) providing greatest diagnostic confidence in detecting/localising NPGLs:

○ Single sequences—FS-T2, CE-FS-T1, pcASL, and TRICKS;
○ Combined sequences—FS-T2 + CE-FS-T1, and pcASL + TRICKS.

A HNPGL of the carotid-body region was defined as a mass splaying the carotid bifurcation (internal carotid artery displaced posteriorly and external carotid artery anteriorly). A vagal paraganglioma was defined as a mass that displaces the internal carotid artery anteriorly. Because of the inherently low signal-to-noise ratio of ASL, pcASL images were visually co-registered with FS-T2 images for interpretation.

### 2.3. Volumetry and Statistics

Tumour volumes were measured on CE-FS-T1 images by one reader (A.R.) using GE Universal Viewer v7.0. Inter-group volume differences were assessed with the Student *t*-test. Inter-observer agreement for lesion detection was calculated with Fleiss’ κ. Observer sensitivity, specificity, and negative predictive values were determined and averaged across readers.

## 3. Results

Twenty-eight patients were enrolled in the study. Eleven had previously diagnosed head-and-neck paragangliomas (HNPGLs)—seven confirmed histologically after surgery and four identified on the basis of a positive family history. The remaining seventeen included eleven subjects undergoing familial screening (eight with HNPGLs and three without) and six patients with surgically confirmed vagal schwannomas. Overall, we analysed eleven vagal and nine carotid paragangliomas (Table 1).

**Figure 1:**
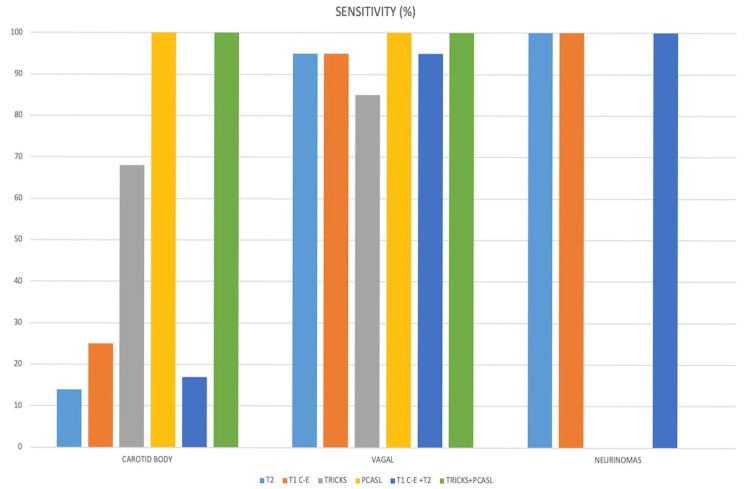
The bar graph shows sensitivity (mean for all readers) for the detection of carotid body tumours, vagal paragangliomas, and neurinomas for each MR imaging technique.

After review of the MRI examinations, inter-reader agreement for NPGL detection was perfect with pcASL alone and with the combined pcASL + TRICKS dataset (κ = 1; Table 2). Pooled sensitivity, specificity, and negative predictive values for each sequence are reported in Table 2 and illustrated in Figure 1.

**Figure 2:**
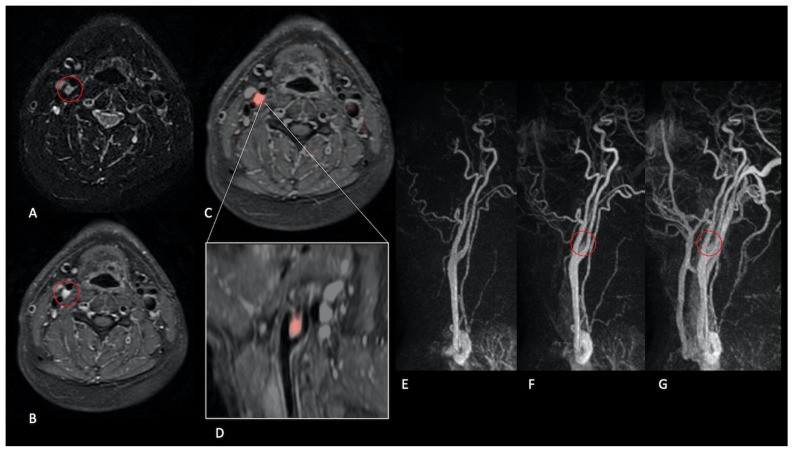
Carotid bifurcation paraganglioma. Patient 14, with a familiar predisposition. MR demonstrates the presence of a small solid lesion at the right carotid bifurcation characterised by T2 hyperintensity (**A**) and homogeneous enhancement after contrast medium administration (**B**) (red circle). Only perfusion ASL imaging, co-registered with fat-suppressed, T1-WI after gadolinium easily shows a marked increase in the TBF parameter within the lesion ((**C**), axial plane and (**D**), sagittal plane). This tissue also demonstrates early enhancement during contrast passage as observed in dynamic contrast-enhanced angiographic acquisitions ((**E**–**G**), red circle).

**Figure 3:**
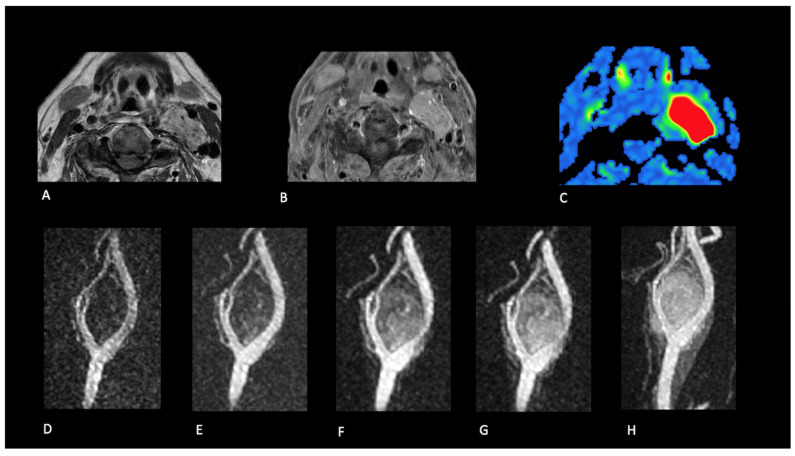
Carotid bifurcation paraganglioma. Patient 18. A solid mass located at the left carotid bifurcation with T2 hyperintensity and flow voids (**A**), homogeneous enhancement after gadolinium (**B**), and a high level of TBF parameter (**C**). This tissue demonstrates early enhancement during contrast passage as observed on dynamic contrast-enhanced angiographic acquisitions (**D**–**H**).

**Figure 4:**
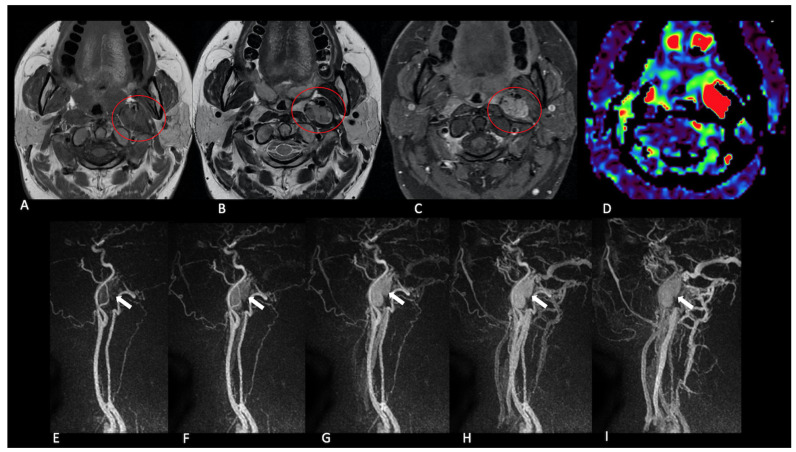
Vagal paraganglioma. Patient 4. A solid mass located in the left lateral-cervical region with anterior displacement of the internal carotid artery. The lesion exhibits a heterogeneous T1 and T2 signal (**A**,**B**) and homogenous enhancement after contrast medium administration (**C**) with some lesional flow-voids (red circle). The lesion presents a pathological increase in the perfusion parameter TBF following ASL acquisition (**D**) and early enhancement during dynamic contrast-enhanced angiographic phases ((**E**–**I**), white arrows).

**Figure 5:**
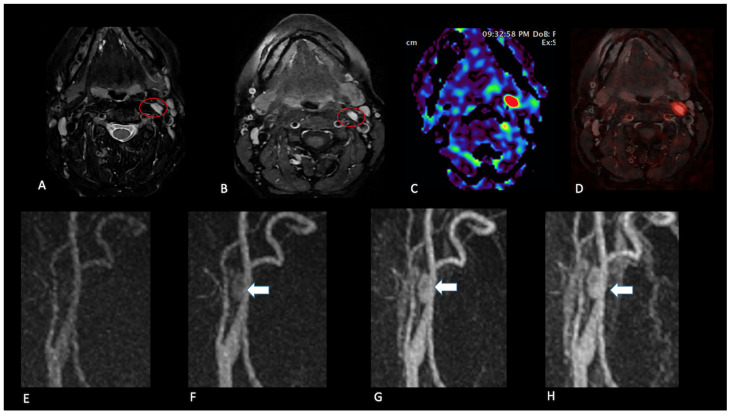
Vagal paraganglioma. Patient 8. A small latero-cervical lesion mimicking a lymph node on FS T2 and T1CE, located close to the left internal carotid artery ((**A**,**B**), red circles). ASL images (**C**) and co-registered images ASL+T1CE (**D**) correctly identified the small paraganglioma, with typical early enhancement during dynamic contrast-enhanced angiographic phases ((**E**–**H**), white arrows).

All previously known NPGLs demonstrated high tumour blood flow (TBF) on pcASL and early arterial enhancement on TRICKS; the same pattern was observed in eight screened relatives (Figure 2, Figure 3, Figure 4 and Figure 5).

Carotid-body paragangliomas were significantly smaller than vagal tumours (mean volume 0.908 cm^3^ ± 0.504 vs. 3.521 cm^3^ ± 0.989; *p* < 0.05).

As shown in Figure 1, the small size of carotid-body lesions limited their visibility on FS-T2, CE-T1, and TRICKS when images obtained from these sequences were evaluated separately. Only pcASL—alone or in combination with TRICKS—consistently detected these tumours.

**Figure 6:**
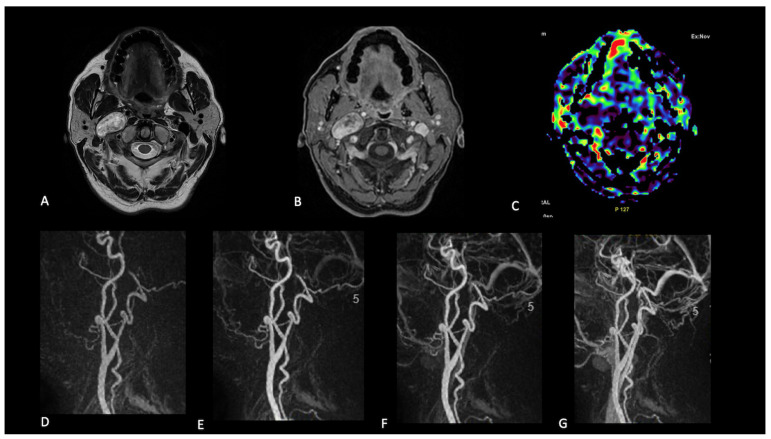
Neck schwannoma. Patient 26. A solid lesion on the right side located close to the internal carotid artery. The lesion showed T2 hyperintensity (**A**) and heterogeneous enhancement after gadolinium (**B**). No TBF increase (**C**) or early enhancement during dynamic contrast-enhanced angiographic phases (**D**–**G**) were evident.

None of the schwannomas exhibited the hyper-vascular pattern described above (Figure 6).

## 4. Discussion

Head-and-neck paragangliomas (HNPGLs) are rare, slow-growing, hyper-vascular tumours that typically appear on conventional T2-weighted MRI as a classic “salt-and-pepper” pattern generated by intratumoural flow voids and focal sub-acute haemorrhage [13]. In our series, however, conventional sequences—including T2-WI, unenhanced, and contrast-enhanced T1-WI, and their combination—showed limited sensitivity, particularly for small lesions.

By contrast, pseudocontinuous arterial spin labelling (pcASL) markedly outperformed 4D contrast-enhanced MR angiography (TRICKS) as well as all conventional sequences. This superiority reflects the intrinsic hyper-vascularity of paragangliomas, which translates into conspicuously elevated tumour blood flow (TBF) on pcASL even when lesions are diminutive.

### 4.1. pcASL: Evidence and Practical Considerations

Recent work on skull-based tumours has confirmed that pcASL reliably separates hyper-vascular lesions such as NPGLs from non-hyper-vascular entities like schwannomas and meningiomas [12]. Quantitative TBF values are significantly higher in paragangliomas than in other lesions, suggesting that pcASL should be incorporated into routine skull-base and neck protocols.

Compared with DSC or DCE perfusion imaging, pcASL requires no contrast agent, is immune to susceptibility artefacts, and involves minimal post-processing [14,15,16,17,18]. Successful acquisition nevertheless depends on three technical choices.

First, the post-labelling delay (PLD) must be short enough for the neck region where the distance between the labelling plane and target tissue is small; values between 1200 and 1800 ms are generally recommended. Our scanner imposes a fixed PLD of 2025 ms, which proved acceptable.

Second, the labelling plane should intersect feeding arteries where they are straight and perpendicular to the plane [19]; we used a rapid phase-contrast neck angiogram to confirm positioning.

Third, image interpretation benefits from co-registration with anatomical T2-WI because the inherently low signal-to-noise ratio of ASL can obscure small structures.

### 4.2. TRICKS and the pcASL + TRICKS Combination

TRICKS captures three-dimensional volumes every 1.8 s during gadolinium passage, dynamically depicting arterial, capillary, and venous phases in a manner reminiscent of digital subtraction angiography (DSA) but without invasiveness [19,20,21,22]. Although its spatial resolution is lower than that of DSA, TRICKS offers wide coverage, high temporal resolution, and reduced motion and venous artefacts compared with non-contrast TOF-MRA.

Earlier work by Van den Berg and colleagues showed that combining unenhanced and contrast-enhanced 3D TOF-MRA is highly effective for detecting small or multicentric paragangliomas, particularly in familial cases [7]. Our findings extend this: fusing pcASL with TRICKS yields the highest diagnostic accuracy across all tumour sizes and locations. The perfusion map highlights hyper-vascularity, while the dynamic angiogram delineates feeding arteries and venous drainage, enabling confident differentiation from other vascular neck masses.

Tiny carotid-body tumours—even those as small as 0.4 cm^3^—often lack the “salt-and-pepper” sign and can mimic lymph nodes on CE-T1-WI. They may also escape detection on TRICKS alone. In our series, only the combined perfusion–angiographic dataset consistently revealed these lesions.

### 4.3. Limitations

The protocol lengthens scanning time, potentially increasing motion artefacts in an anatomically challenging region. Moreover, the study cohort was modest; larger prospective investigations are needed to confirm our observations.

## 5. Conclusions

pcASL and TRICKS provide complementary information—perfusion and dynamic vascular anatomy—that together allow reliable identification of hyper-vascular neck lesions. Integrating both sequences into standard head-and-neck MRI protocols should be considered essential for the accurate characterisation of paragangliomas.

## Figures and Tables

**Table 1:** Patient demographics.

Patient No.	Age (Years)	Sex	Lesion Type	Location	Volume (cm^3^)	Treatment	Surgery	Recurrence	Radiotherapy	Familial Predisposition
1	26	F	PARAGANGLIOMA	VAGAL	4.112	YES	YES	YES	NO	YES
2	53	M	PARAGANGLIOMA	CAROTID	0.659	YES	YES	YES	NO	YES
3	38	F	PARAGANGLIOMA	VAGAL	4.221	YES	YES	NO	NO	NO
4	58	M	PARAGANGLIOMA	VAGAL	3.558	YES	YES	YES	NO	YES
5	53	F	PARAGANGLIOMA	CAROTID	0.456	YES	YES	NO	NO	NO
6	45	F	PARAGANGLIOMA	VAGAL	5.656	YES	YES	NO	NO	NO
7	48	F	PARAGANGLIOMA	VAGAL	3.995	NO	NO	NO	NO	YES
8	62	F	PARAGANGLIOMA	VAGAL	1.287	NO	NO	NO	NO	YES
9	79	F	PARAGANGLIOMA	VAGAL	2.457	NO	NO	-	NO	YES
10	50	F	PARAGANGLIOMA	CAROTID	0.943	NO	NO	-	NO	YES
11	59	M	PARAGANGLIOMA	VAGAL	4.956	YES	YES	YES	NO	YES
			PARAGANGLIOMA	VAGAL	4.821	NO	NO	-	NO	YES
12	74	F	PARAGANGLIOMA	CAROTID	0.599	NO	NO	-	NO	YES
13	66	F	PARAGANGLIOMA	VAGAL	6.955	NO	NO	-	NO	YES
14	54	F	PARAGANGLIOMA	CAROTID	0.541	NO	NO	-	NO	YES
15	79	M	PARAGANGLIOMA	CAROTID	1.788	NO	NO	-	NO	YES
16	83	F	PARAGANGLIOMA	VAGAL	4.218	NO	NO	-	NO	YES
17	54	F	PARAGANGLIOMA	VAGAL	2.659	NO	NO	-	NO	YES
			PARAGANGLIOMA	CAROTID	0.398	NO	NO	-	NO	YES
18	65	M	PARAGANGLIOMA	CAROTID	4.266	NO	NO	-	NO	YES
19	59	F	PARAGANGLIOMA	CAROTID	1.522	NO	NO	-	NO	YES
20	35	M	-	-	-	-	-	-	-	YES
21	29	M	-	-	-	-	-	-	-	YES
22	44	M	-	-	-	-	-	-	-	YES
23	48	F	SCHWANNOMA	NECK	6.544	YES	YES	-	NO	-
24	55	F	SCHWANNOMA	NECK	4.877	YES	YES	-	NO	-
25	68	F	SCHWANNOMA	NECK	6.764	YES	YES	-	NO	-
26	62	M	SCHWANNOMA	NECK	4.832	YES	YES	-	NO	-
27	71	F	SCHWANNOMA	NECK	4.521	YES	YES	-	NO	-
28	59	M	SCHWANNOMA	NECK	5.961	YES	YES	-	NO	-

**Table 2:** Concordance test and cumulative reader’s sensitivity, specificity, and negative predictive values.

	Fleiss k	Sensitivity	Specificity	NPV
**T2**	0.611	0.6 (0.14–0.95)	0.3 (0.12–0.62)	0.85
**C-E**	0.766	0.71 (0.25–0.95)	0.28 (0.14–0.69)	0.87
**TRICKS**	0.587	0.82 (0.68–0.94)	1 (1.00–1.00)	0.93
**PCASL**	1	1 (1.00–1.00)	1 (0.00–1.00)	1
**T2+C-E**	0.572	0.6 (0.17–0.95)	0.32 (0.14–0.71)	0.86
**TRICKS+PCASL**	1	1 (1.00–1.00)	1 (1.00–1.00)	1

## Data Availability

The data are available from the corresponding author, A.R. (Andrea Romano), upon reasonable request.
